# Application of Nanotechnology and Phytochemicals in Anticancer Therapy

**DOI:** 10.3390/pharmaceutics16091169

**Published:** 2024-09-05

**Authors:** Jin Hee Kim, Boluwatife Olamide Dareowolabi, Rekha Thiruvengadam, Eun-Yi Moon

**Affiliations:** 1 Department of Integrative Bioscience & Biotechnology, Sejong University, Seoul 05006, Republic of Korea; boluwatifedareowolabi@gmail.com (B.O.D.); eunyimoon@sejong.ac.kr (E.-Y.M.); 2 Center for Global Health Research, Saveetha Institute of Medical and Technical Sciences (SIMATS), Saveetha Medical College, Saveetha University, Chennai 600077, India; reklak4@gmail.com

**Keywords:** cancer, postoperative anticancer therapy, nanotechnology, nanomedicine, phytochemicals

## Abstract

Cancer is well recognized as a leading cause of mortality. Although surgery tends to be the primary treatment option for many solid cancers, cancer surgery is still a risk factor for metastatic diseases and recurrence. For this reason, a variety of medications has been adopted for the postsurgical care of patients with cancer. However, conventional medicines have shown major challenges such as drug resistance, a high level of drug toxicity, and different drug responses, due to tumor heterogeneity. Nanotechnology-based therapeutic formulations could effectively overcome the challenges faced by conventional treatment methods. In particular, the combined use of nanomedicine with natural phytochemicals can enhance tumor targeting and increase the efficacy of anticancer agents with better solubility and bioavailability and reduced side effects. However, there is limited evidence in relation to the application of phytochemicals in cancer treatment, particularly focusing on nanotechnology. Therefore, in this review, first, we introduce the drug carriers used in advanced nanotechnology and their strengths and limitations. Second, we provide an update on well-studied nanotechnology-based anticancer therapies related to the carcinogenesis process, including signaling pathways related to transforming growth factor-β (TGF-β), mitogen-activated protein kinase (MAPK), phosphatidylinositol 3 kinase (PI3K), Wnt, poly(ADP-ribose) polymerase (PARP), Notch, and Hedgehog (HH). Third, we introduce approved nanomedicines currently available for anticancer therapy. Fourth, we discuss the potential roles of natural phytochemicals as anticancer drugs. Fifth, we also discuss the synergistic effect of nanocarriers and phytochemicals in anticancer therapy.

## 1. Introduction

Cancer is well recognized as a leading cause of mortality. Approximately 19.3 million new cancer cases and 10 million cancer-caused deaths in 2020 worldwide were reported [1,2]. Among the new cases, breast cancer was the most frequent, followed by lung, colon, prostate, skin, and stomach cancers [2]. Although surgery tends to be the primary treatment option for many solid cancers, cancer surgery has been well documented to be a risk factor for metastatic diseases and recurrence in many clinical and experimental studies [3]. The perioperative phase of cancer surgery offers a treatment window against lingering malignant illness and is critical for assessing the risk of postoperative metastatic diseases [3]. For this reason, a variety of medications has been adopted for the postsurgical care of patients with cancer [4]. However, conventional medicines have shown major challenges such as drug resistance, a high level of drug toxicity, and different drug responses due to tumor heterogeneity [5,6]. Recently, a nanomedicine-based therapeutic strategy was suggested to be a promising alternative to improve the efficiency and selectivity of anticancer drugs in anticancer therapy [7]. Because nanomedicine-based therapeutic drugs help target tumor sites, these drugs can control the local and systemic releases of medicines, resulting in enhanced therapy efficacy, reduced toxicity, and improved patient outcomes [5,7,8]. In particular, tumor-targeted nanoparticle (NP)-based anticancer therapy is considered an extensive and favorable era in cancer biology [9].

Many proteins are involved in the carcinogenesis process, including signaling pathways related to transforming growth factor-β (TGF-β), mitogen-activated protein kinase (MAPK), phosphatidylinositol 3 kinase (PI3K), Wnt, poly(ADP-ribose) polymerase (PARP), Notch, and Hedgehog (HH) [10]. Because inhibitors against the proteins involved in carcinogenesis can specifically target molecular mechanisms related to the promotion of cancer growth, metastasis, and carcinogenesis-related inflammatory processes, conjugation of the inhibitors in chemotherapy can be effective in destroying cancer cells and preventing metastasis [9]. Especially, NPs based on the inhibitors of carcinogenesis-related proteins provide better anticancer drug efficacy, overcoming major constraints in conventional chemotherapy such as low bioavailability, many side effects, and poor solubility [5,9].

Phytochemicals are considered anticancer agents because of their inhibitory roles against inflammation and postsurgical recurrence of cancer and metastasis [11,12,13]. Natural phytochemicals have few side effects as well as anticancer effects [14,15]. However, there is limited evidence in relation to the application of phytochemicals in cancer treatment, particularly focusing on nanotechnology. Therefore, in this review, we discussed carcinogenesis inhibitor-based drug delivery strategies using nanotechnology in anticancer therapy and the potential role of natural phytochemicals as anticancer agents.

## 2. Drug Delivery Strategies Using Nanotechnology

A variety of anticancer drugs can be used in anticancer therapy. Notably, drug carriers play a pivotal role in anticancer therapy by improving the delivery and efficacy of therapeutic agents while minimizing side effects [5,9]. Many types of drug carriers, including NP, nanocapsules, nanoemulsions, and hydrogels, can be used in cancer therapy [16,17,18,19,20,21,22,23,24,25,26,27,28,29,30,31,32,33,34,35,36,37,38,39,40,41,42,43,44,45,46,47]. Moreover, these nanotechnology-based anticancer drugs can be used to prevent carcinogenesis through several signaling pathways. Therefore, in this Section, we introduce the drug carriers used in advanced nanotechnology and summarize anticancer strategies focusing on nanotechnology-based anticancer therapy by carcinogenesis signaling pathways related to TGF-β, MAPK/PI3K/Wnt, PARP, Notch/HH, and others.

### 2.1. Drug Carriers Used in Advanced Nanotechnology

**Figure 1:**
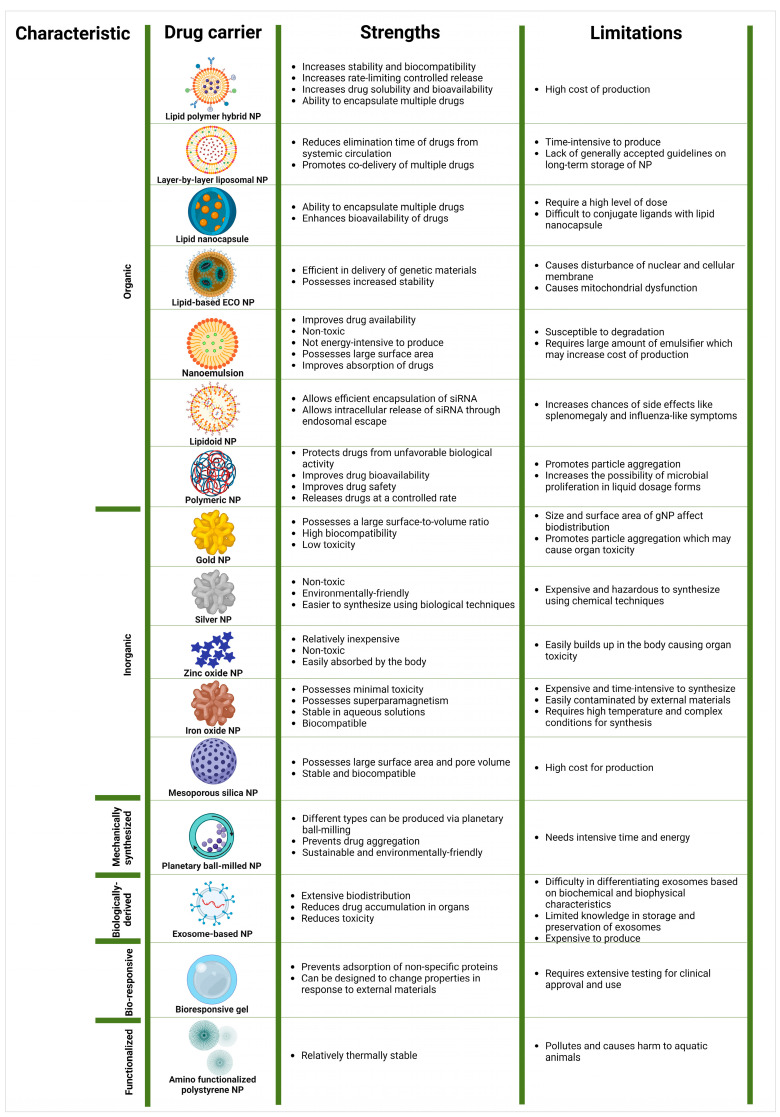
Drug carriers used in advanced nanotechnology and their strengths and limitations [16,17,18,19,20,21,22,23,24,25,26,27,28,29,30,31,32,33,34,35,36,37,38,39,40,41,42,43,44,45,46,47]. NP, nanoparticle.

Figure 1 shows drug carriers used in advanced nanotechnology and their strengths and limitations.

The lipid polymer hybrid NP is a new form of hybrid NP, created for the targeted transportation of chemotherapeutic medicines to tumor cells [16]. It is made up of three layers, a polymer core where the drugs are contained, a lipid monolayer that surrounds the polymeric core, and a lipid polyethylene glycol (PEG) layer on which special targeting moieties can be attached [16]. This NP has increased stability and biocompatibility, increased drug half-lives, and increased rate-limiting controlled release [17].

Layer-by-layer liposomal NPs are formulated to combine the advantages of designing multilayer structures with nanometer precision with the advantage of liposomes [18]. Because of the multilayer, the elimination time of drugs from the systemic circulation is reduced, thus promoting effective drug delivery [18]. However, this multilayer technique is time-consuming, making production on a large-scale level difficult [19].

A lipid nanocapsule is a carrier system made up of an oily hydrophobic core surrounded by a combination of PEGylated surfactants and phospholipids [20]. Lipid nanocapsules are more efficient in encapsulating drugs for delivery when compared to other conventional NPs [21]. They can also encapsulate multiple drugs at once and enhance the bioavailability of encapsulated drugs [21]. Lipid nanocapsules, however, require a high-level dose for their function [22]. Conjugation of ligands with lipid nanocapsules has also proven to be challenging [22].

Lipid-based ECO NP is a multifunctional drug carrier that is effective at mediating gene silencing [23]. The ECO structure promotes the stability of the nanocarrier [23] and is quite efficient in the delivery of genetic materials [24]. Cationic lipids, however, have several adverse effects including disturbance of nuclear and cellular membranes and releasing degrading enzymes from lysosomes [25].

A nanoemulsion is a colloidal system of 10 to 1000 nm in size [26]. It is made up of solid spheres with lipophilic and amorphous surfaces [26]. A nanoemulsion has several strengths as a carrier for drugs [26]. It is non-toxic and not energy-intensive, improves the bioavailability and solubility of the drug, and provides greater surface area for improved absorption of drugs [26]. However, it is susceptible to degradation [27].

Lipidoids are synthetic cationic lipids that have secondary and tertiary amine functions and efficient interactions with anionic siRNA molecules [28]. Lipidoid NPs are capable of delivering siRNA [29]. Ionizable cationic lipidoids allow improved encapsulation of siRNA and its intracellular release [29].

Polymeric NPs with a size ranging from 1 to 1000 nm can contain drugs within or on their surface [30]. They have several advantages that prove their potential to effectively deliver drugs. They protect the drugs they carry from biological activity in the environment, thus improving their bioavailability and drug safety [30]. They also have the ability to release drugs at a controlled rate [30]. Despite all these advantages, polymeric NPs are still limited in large-scale production due to particle aggregation, premature release of drugs, and microbial proliferation in liquid dosage forms [30].

A gold NP (gNP) can be developed with a synthetic method that involves treating hydrogen tetrachloroaurate and citric acid [31]. A gNP has a low toxicity, high biocompatibility, and large surface-to-volume ratio [32]. However, the size and surface charge of gNPs affect their biodistribution leading to aggregation in a few organs, which may promote toxicity [32].

A silver NP (sNP) can be synthesized via several methodologies including biological and chemical methods [33]. sNPs have the advantage of being non-toxic, faster to synthesize, and environmentally friendly, especially when synthesized via the biological method [33]. It is expensive and hazardous to synthesize via the chemical method [34].

Zinc oxide NPs are one of the most popular metal NPs used in anticancer medication [35]. Zinc oxide NPs can be synthesized through chemical precipitation using a highly purified zinc forerunner and a precipitator [35]. These NPs are relatively inexpensive, non-toxic, and easily absorbed by the body [35]. However, their major limitation is that they can easily build up in the body, resulting in organ toxicity [36].

Iron oxide NPs are made from iron oxide via physical, chemical, or biological techniques [37]. Iron oxide NPs are one of the most preferred NPs for drug delivery because they possess a number of advantages. They cause minimal toxicity, are stable in aqueous solutions, possess superparamagnetism, and are biocompatible [38]. However, they are hard to produce; in particular, the physical technique requires expensive and complex machines [37]. This makes it difficult to control the size of the NPs [37]. Moreover, the chemical technique leads to it being easily contaminated by external materials as well as requiring very high temperatures and complex conditions for its production [37].

Mesoporous silica NPs are inorganic NPs with a size that ranges from 30 to 300 nm [39]. They possess several advantages including a large surface area and large pore volume, and they are quite stable and biocompatible [40]. They are quite difficult to produce in an industrial setting due to their high cost [40].

Planetary ball milling used for the synthesis of NPs involves rotating a vial in a planet-like motion to reduce the size of large crystals [41]. This technique mixes drug powder with a dispersion medium and a stabilizer, which helps to prevent drug aggregation [42]. Planetary ball-milled NPs (PBM-NPs) are sustainable and environmentally friendly as planetary ball milling adopts sustainable materials as precursors for NPs [41]. The planetary ball-milling process, however, is both time and energy-consuming [42].

Exosomes are natural vesicular structures released from cells and have sizes ranging from 30 to 150 nm [43]. Exosome-based NPs may have extensive biodistribution and reduced accumulation in organs, resulting in reduced toxicity [43]. However, there are some difficulties in differentiating exosomes based on biochemical and biophysical characteristics [43]. Large-scale production of exosome-based NPs is expensive as it requires a large number of manufacturing devices [43].

A bioresponsive gel is a class of highly hydrated biomaterials [44]. It provides an environment that is semi-wet and suitable for biological interactions on a molecular level [44]. It also provides an inert surface that prevents the adsorption of non-specific proteins [44]. It can be designed to change properties in response to external materials [44]. Its major limitation is that it needs extensive testing to explore the effects of its component parts in the body [44,45]. This could be time and money-intensive [45].

An amino-functionalized polystyrene NP is a class of NPs based on a polymer backbone with amino moieties [46]. Polystyrene NPs are relatively thermally stable; however, they have high toxicity potentials, and are known to pollute and cause harm to aquatic animals [47].

### 2.2. TGF-β Signaling-Based Nanotherapies

The TGF-β superfamily consists of ligand proteins, such as bone morphogenetic proteins, activins, and proteins related to their associated receptors, which allow the translocation of Smad proteins into the nucleus for transcription of tumor progression and metastasis-related genes [48]. The TGF-β signaling pathway plays a pivotal part in various processes, including the proliferation and migration of cells [49]. The anticancer effects of TGF-β signaling-based nanotherapies are listed in Table 1.

Small interfering RNA (siRNA) to integrin β3 via lipid ECO-based NPs (ECO/siβ3) can effectively silence integrin β3 expression, restore TGF-β-mediated cytostasis, decrease TGF-β-mediated epithelial–mesenchymal transition (EMT) and invasion, and suppress three-dimensional organoid growth in triple-negative breast cancer (TNBC) [50]. Thus, it acts as a promising therapeutic regimen to combat TNBC [50]. Poly-N-(2-hydroxypropyl) methacrylamide (pHPMA)-coated hybrid NPs with modified lipid polymer co-loaded with cryptotanshinone (S/C-pW-LPNs) and silibinin have been evaluated to identify their anti-metastasis efficacy in a mouse model with breast cancer [51]. These NPs effectively decreased microenvironment biomarkers such as TGF-β1, matrix metalloprotease 9 (MMP-9), and platelet and endothelial cell adhesion molecule 1 (PECAM1, also called CD31) related to metastasis, indicating that the NPs are effective nanocarriers of an oral drug to inhibit metastasis of breast cancer to the lung [51]. Zinc oxide NPs can effectively decrease cell proliferation and migration, enhance apoptotic bodies, alter cell cycle distribution, and decrease the synthesis of MMP-9 and TGF-β in murine photoreceptor-derived cells [52]. The codelivery of TGF-β1 with silver NPs can effectively modulate the immune response and significantly reduce the severity of inflammatory diseases such as autoimmune encephalomyelitis and multiple sclerosis by programming antigen-presenting cells to induce a more efficient tolerance [53]. In TGF-β-stimulated fibroblasts, the NPs releasing siRNAs targeting heat shock protein 47 (HSP47) effectively reduced profibrotic markers such as NADPH oxidase 4, collagen type I, and alpha-smooth muscle actin in a fibrosis model [54]. This evidence supports that TGF-β signaling-based nanotherapies can improve the anticancer effect.

### 2.3. MAPK/PI3K/Wnt Signaling-Based Nanotherapies

The MAPK pathway is composed of several important signaling cascades, including rat sarcoma (RAS), rapidly accelerated fibrosarcoma (RAF), mitogen-activated protein kinase (MEK), and extracellular signal-regulated kinase (ERK called MAPK) [55]. Extracellular signals, such as growth factors and cytokines, activate tyrosine kinase receptors, inducing MAPK signaling [55]. The ERK is stimulated by various inflammatory mediators, including cytokines, chemokines, and lipopolysaccharides [56] and activated ERK stimulates proinflammatory cytokines, indicators of carcinogenesis [57]. The PI3K pathway is also dysregulated in approximately 30% of cancers [58]. PI3K, a heterodimer, consists of catalytic and regulatory subunits [58]. Activated PI3K phosphorylates phosphatidylinositol 4,5-bisphosphate (PIP2) to make phosphatidylinositol 3,4,5-trisphosphate (PIP3), which can activate pyruvate dehydrogenase kinase 1 and sequentially phosphorylate AKT serine/threonine kinase 1 (AKT), which inhibit the transcription of tumor suppressor genes [58]. Dysregulation of the canonical Wnt pathway signaled by Wnt and β-catenin is also crucial for cancer progression [59]. The anticancer effects of MAPK/PI3K/Wnt signaling-based nanotherapies are listed in Table 2.

The siRNA to RAF or AKT could be loaded in cationic nanoliposomes, and the siRNA-loaded nanoliposomes selectively target melanoma tumor cells as well as early melanocytic lesions, resulting in the prevention of melanoma metastasis [60]. Sorafenib is an inhibitor of the receptor tyrosine kinase that plays a pivotal role in the MAPK/PI3K signaling cascade for tumor development and metastasis in various human cancers, including metastatic liver, gastrointestinal stromal, hypernephroma, and colorectal cancers [61]. Sorafenib-loaded lipid-based nanosuspensions have greater aqueous solubility, higher encapsulation efficiency, and improved bioavailability compared with free Sorafenib, which results in better treatment efficacy by reducing proliferation of tumor cells and enhancing reorganization of the MAPK cascade in glioblastoma therapy [61].

The anticarcinogenic effect of amino-functionalized polystyrene (NH2-PS) NPs was compared with that of amino-functionalized silica (NH2-Si) or hydroxyl-functionalized silica (OH-Si) NPs in hepatocellular carcinoma (HCC) Huh7 and HepG2 cell lines [62]. At the molecular level, NH2-PS NPs obstructed mammalian target of rapamycin (mTOR) signaling, damaged the mitochondrial membrane, and enhanced lysosomes that precede cell death [62]. Generally, the NH2-PS NPs were more effective than the NH2-Si NPs [62]. Iron oxide can stimulate lysosome dysfunction and change the subcellular localization of p53 and mTOR, which can affect the autophagic flux [63]. The treatment of the chemotherapeutic drug cisplatin inhibiting DNA replication with anti-human epidermal growth factor receptor 2 (HER2) antibody-conjugated and autophagy inhibitory microRNA (MIR376B)-loaded superparamagnetic iron oxide NPs enhanced anticancer treatment efficiency in both xenograft nude mice with breast cancer and HER2-positive breast cancer cells [64]. Everolimus, a mTOR inhibitor, is used as an immune suppressor [65]. When p53-encoding synthetic mRNA was delivered using NP technology, it effectively restored tumor suppressor p53 in tumor sites and resulted in tumor cells sensitive to everolimus [66]. Thus, co-targeting p53 and the mTOR signaling pathway can effectively exhibit an antitumor effect in HCC and non-small cell lung cancer (NSCLC) [66]. Bioreducible polymer was used to encapsulate siRNA inhibiting mTOR and it exhibited strong potential to deliver siRNA to lung cancer cells [7]. PI3K inhibitors entrapped in supramolecular nanoassemblies with 1,2-distearoyl-sn-glycero-3-phosphoethanolamine (DSPE)-PEG and L-α-phosphatidylcholine could induce an anticancer effect by increasing the phosphorylation of mTOR and AKT, thus resulting in increased antitumor efficacy and longevity [67]. The overactivation of PI3K/mTOR signaling has been observed in non-Hodgkin’s lymphoma [68]. BEZ235, a dual PI3K and mTOR inhibitor, has been found to be an effective suppressor of lung cancer [69]. When dibenzocyclooctyne-functionalized anti-Lym1 and anti-CD20 antibodies were used in an NP-based drug delivery system for delivering BEZ235 to lymphoma cells, this system improved antitumor activity of BEZ235 both in vivo and in vitro via inhibiting the PI3K/mTOR signaling pathway [68]. When AZD6244 (selumetinib, an allosteric inhibitor of MEK1/2) and PX-866 (a PI3K inhibitor) were layer-by-layer co-encapsulated in a cancer-targeting nanoscale therapeutic formulation, they effectively blocked lobular carcinoma in xenograft-bearing NCR nude mice [70]. Chrysophanol gNPs have been evaluated against human LNCaP prostate cancer cells [71]. Chrysophanol gNPs were found to reduce histone deacetylase activities and halt the cell cycle in the sub-G phase via the inactivation of AKT and upregulation of AMP-activated protein kinase (AMPK) and sequentially controlling the activity of mTOR, and finally inhibit prostate cancer cell growth [71,72]. PH-427, an AKT/pyruvate dehydrogenase kinase 1 (PDK1) inhibitor, is encapsulated into poly(lactic-co-glycolic acid) (PLGA) NPs (PH-427-PLGA-NPs), and treatment with PH-427-PLGA-NPs reduced the tumor size in a MiaPaCa-2 pancreatic cancer model, indicating that NPs can be efficient drug carriers targeting pancreatic cancer that harbors *RAS* mutations [73].

Sorafenib-loaded PEG-PLGA NPs modified with the antibody hGC33 to glypican-3 (GPC3) can effectively target GPC3-positive HCC cells by inhibiting the Wnt pathway, downregulating cyclin D1 expression, inhibiting EMT, and inactivating the RAS and RAF on MAPK signaling pathway [74]. All the above-mentioned evidence supports that MAPK/PI3K/Wnt signaling-based nanotherapies can improve the anticancer effect.

**Table 2:** MAPK/PI3K/Wnt signaling-based nanotherapies.

Nanomedicine Name	Drug in Nanomedicine	Delivery System	Target Cancer	Experimental Model	Effect of Nanomedicine on Cancer	Ref.
Nanoliposomal siRNA	Small interfering RNA targeting B-Raf with V600E and AKT3	Cationic nanoliposomes	Melanoma	Human melanoma cell lines; human fibroblasts	Decreased expression of B-Raf with V600E and AKT; decreased melanoma by 65%	[60]
SFN-LNC	Sorafenib	Lipid nanocapsules	Glioblastoma	Human U87MG glioblastoma cell lines; mice with orthotopic U87MG human glioblastoma xenografts	Inhibited in vitro angiogenesis; decreased glioblastoma cell viability; decreased proliferating cells in tumor	[61]
NH2-PS and NH2-Si NP	Amino-functionalized polystyrene and biodegradable silica	Amino-functionalized polystyrene NPs and amino-functionalized silica NPs	HCC	HCC cell lines	NH2-PS NPs trigger death of Huh7 and HepG2 cells by obstructing mTOR signaling and inducing lysosomal destabilization; NH2-Si enhances cell proliferation by activating mTOR signaling	[62]
Iron oxide-based NPs	Magnetite core coated with carboxymethyldextran shell	Green fluorescent labeled iron oxide NPs (nano-screenMAG-CMX) and non-fluorescent magnetic particles (fluidMAG-MX)	Hepatoblastoma	Hepatic cell line (HepG2)	Induced lysosomal dysfunction; altered subcellular localizations of pmTOR and p53 proteins	[63]
SPION NPs	MicroRNA (MIR376B)	AGO2 conjugated and anti-HER2 labeled SPIONs (SP-AH)	Breast cancer	HER2-positive breast cancer cell lines; xenograft nude mice model of breast cancer	Blocked autophagy; increased the efficacy of anticancer treatment	[64]
Supramolecular NPs	PI103 and PI828	Supramolecular nano-assembly using L-α-phosphatidylcholine, and DSPE-PEG [1,2-distearoyl-sn-glycero-3-phosphoethanolamine-N-[amino(polyethylene glycol)]	Breast and ovarian cancers	4T1 breast cancer and K-Ras (LSL/+)/PTEN (fl/fl) ovarian cancer models	Temporally sustained inhibition of phosphorylation of AKT, mTOR, S6K, and 4EBP in vivo; increased antitumor efficacy; abrogated insulin resistance	[67]
NP-based pre-targeted system for the therapeutic delivery of BEZ235	BEZ235	Azide-functionalized BEZ235-encapsulated NPs	Non-Hodgkin’s lymphoma	Lymphoma cell lines	Improved in vivo and in vitro antitumor activity of BEZ235 by inhibiting the PI3K/mTOR pathway	[68]
Lbl NP	AZD6244; PX-866	Tumor targeting nanoscale drug formulation (layer-by-layer NPs)	TNBC; RAS-mutant lung tumor	Cancer cell lines (MDA-MB-231, Hep G2, KP7B, and OVCAR-3 cells)	Caused cytotoxicity in both the TNBC cell line and RAS-mutant lung tumor cell line; blocked tumor-specific phosphorylation of ERK and AKT	[70]
Gold-chrysophanol NPs	Chrysophanol	PLGA NPs	Prostate cancer	LNCap prostate cancer cells	Induced apoptosis; increased ROS production; caused DNA damage; expressed differentially pro- and anti-apoptotic proteins; reduced tumor volume and weight	[71]
PLGA NPs	PH-427	PLGA NPs	Pancreatic cancer	MiaPaCa-2 pancreatic cancer model with mutant K-ras	Improved drug delivery and therapeutic efficacy against pancreatic cancer with mutant K-ras	[73]
hGC33-modified NPs (hGC33-SFB-NP)	Sorafenib	Polyethylene glycol-b-PLGA polymer NPs	HCC	In vivo model of liver cancer	Inhibited growth and progression of liver cancer by targeting GPC3+ HCC cells; attenuated HCC cell migration; inhibited epithelial–mesenchymal transition	[74]

AKT, AKT serine/threonine kinase 1; GPC3, glypican-3; HCC, hepatocellular carcinoma; HER2, human epidermal growth factor receptor 2; HSP47, heat shock protein 47; NH2-PS, amino-functionalized polystyrene; NH2-Si, amino-functionalized silica; NP, nanoparticle; PLGA, poly(lactic-co-glycolic acid); TGF-β, transforming growth factor-beta; TNBC, triple-negative breast cancer.

### 2.4. PARP Signaling-Based Nanotherapies

PARP is a family of proteins crucial for DNA repair and apoptosis [75]. Upon sensing damage, PARP is activated via its phosphorylation by AKT to recruit the machinery required for DNA repair [76,77]. Because activated PARP reduces the death of cancer cells in cancer therapy, the inhibition of PARP activation can be very important in cancer therapy. The anticancer effects of PARP signaling-based nanotherapies are listed in Table 3. A PARP inhibitor (PARPi), Talazoparib, has been encapsulated in the bilayer of a nanoliposome to develop nanoTalazoparib [78]. In *BRCA*-deficient breast cancer mice, nanoTalazoparib enhanced the survival of mice, induced DNA damage, led to cell cycle arrest, and inhibited cell proliferation by modulating the immune cells [78]. When radiation-resistant cells and tumors derived from a *p53/phosphatase* and *tensin homolog* (*PTEN*)-deficient mouse model of advanced prostate cancer were treated with a lipid-based nanoformulation of Olaparib (nanoOlaparib), it made radiation-resistant tumors without *BRCA* mutations radiosensitive [79]. The newly developed fluorescence-labeled PARPi-encapsulated nanoemulsion (PARPi-FL) had a prolonged circulation time [80]. The longer half-life indicates the pharmacokinetic benefits of nanoemulsions as nanocarriers, thereby confirming the importance of PARPi-FL as an imaging agent targeting PARP in small cell lung cancer [80]. The overexpression of the Rad6 protein in breast cancer during the aggressive stage contributes to DNA damage tolerance [81]. The inhibitor for Rad6 (SMI#9)-conjugated gNP (SMI#9-gNP) can be endocytosed by mesenchymal TNBC cells, resulting in cytotoxicity [82]. In addition, the co-administration of SMI#9-gNP with cisplatin demonstrated a synergistic effect by enhancing cisplatin sensitivity without causing damage to normal breast cells [82]. SMI#9 released from gNPs causes cell death by inducing mitochondrial dysfunction and PARP1 hyperactivation, finally acting as an effective formulation that specifically targets chemo-resistant TNBC cells [82]. Liposomal NPs co-loaded with PARPi and cisplatin have been developed layer-by-layer using electrostatics with a hyaluronic acid layer at the terminal, which facilitates targeting the CD44 receptor, and thus could selectively target ovarian cancer [83]. The liposomal NPs showed increased blood circulation time with significantly lower systemic toxicity, minimized tumor metastasis, and increased the survival of CD44-expressing female nude mice, thus improving their therapeutic efficacy against high-grade serous ovarian cancer [83]. NP-mediated delivery of siRNA targeting PARP1 in mouse ovarian cancer models significantly inhibited cell proliferation, induced apoptosis, and prolonged the survival of mice with tumors [84]. In one study, Veliparib (a PARPi) and methylene blue (a photosensitizer) co-encapsulated in PLGA NPs presented reduced cytotoxicity of normal cells in the dark with reduced viability of cancer cells [85]. This result indicates that the co-encapsulation of Veliparib and methylene blue could be an important strategy to improve photodynamic therapy [85]. Linalool is a monoterpene compound, and when gNPs conjugated with linalool and the CALNN peptide were treated to ovarian SKOV-3 cancer cells, apoptosis was induced by activating p53 and caspase-8 [86]. This indicates that ovarian cancer could be suppressed by NPs by inducing apoptosis via extrinsic and intrinsic pathways [86]. This evidence supports that PARP signaling-based nanotherapies can improve the anticancer effect.

### 2.5. Notch/HH Signaling-Based Nanotherapies

Notch signaling is also an oncogenic signaling pathway crucial for cancer invasiveness and progression [87]. It is also involved in EMT stimulation [88]. The HH signaling is also known to be pivotal for cancer stem cell maintenance, chemoresistance, and radioresistance via inducing transcription of oncogenes [89]. Anticancer effects of Notch/HH signaling-based nanotherapies are listed in Table 4.

Mesoporous silica NPs with glucose moieties and γ-secretase inhibitors, strong interceptors to Notch signaling, have been tested against breast cancer cells [90]. These NPs were effectively internalized and decreased the population of malignant stem cells [90]. α-Mangostin-encapsulated PLGA NPs (Mang-PLGA-NPs) have been developed and assessed in colorectal cancer cells [91]. Mang-PLGA-NPs inhibited colorectal cancer cell viability, colony formation, and EMT, enhanced programmed cell death, and inhibited the cancer stem-like cell population by suppressing Notch signaling components (such as Notch-1, Notch-2, DLL4, and Jagged 1), Hes-1, and γ-secretase complex protein, indicating that Mang-PLGA-NPs could be used to treat and prevent colorectal cancer [91].

A PBM-NP has been developed using thymoquinone (TQ), a natural polysaccharide, and A10, an RNA aptamer that is bound to prostate-specific membrane antigen [92]. When the A10-coated PBM-NP with TQ (A10-TQ-PBM-NP) was used to treat two prostate cancer cell lines LNCaP-R and C4-2B-R, which are resistant to docetaxel with high HH expression, the A10-TQ-PBM-NP was highly effective in inhibiting the HH signaling pathway and sequentially suppressing prostate cancer progression [92]. Another study engineered a nano-size molecule HH signaling inhibitor (nanoHHi)-containing polymeric NPs using PLGA conjugated with PEG [93]. Encapsulated nanoHHi effectively decreased pancreatic cancer cell proliferation, and the use of nanoHHi together with gemcitabine impeded the growth of orthotopic xenografted Pa03C pancreatic cancer better than use of gemcitabine alone [93]. In addition, engineered dual-targeting biomimetic NPs containing LDE225 (an inhibitor to Sonic hedgehog (Shh)) and apolipoprotein A1 (an anti-CD15) functioned as an effective and stable drug carrier [94]. They were able to cross the blood–brain barrier (BBB) and deliver drugs to the cancer stem-like cells of medulloblastoma with a high Shh level, indicating that the NP could be used as a potent and effective nanomedicine to treat medulloblastoma with a high Shh level [94]. The medulloblastoma with a high Shh level can be also targeted by biomimetic high-density lipoprotein (HDL) NPs that bind to the HDL receptor (scavenger receptor type B-1, SCARB1), resulting in the depletion of cholesterol levels in cancer cells and thus effectively blocking the proliferation of medulloblastoma cells and colony formation [95]. Spherical nucleic acid NPs wrapped by a polyethylenimine shell target the transcription factor Gli1, which plays a role in the HH signaling pathway required for glioma stem cell maintenance [96]. These Gli1-targeted NPs bind to scavenger receptors on glioblastoma cells and induce dynamin-dependent and caveolae-mediated endocytosis [96]. The Gli1-targeted NPs can inhibit the tumor-promoting HH pathway and its downstream target genes, thereby alleviating drug resistance and glioblastoma recurrence [96]. The effect of nanoHHi alone or in combination with Sorafenib has been tested in HCC cell lines, and it significantly inhibited the proliferation, invasion, systemic metastasis, as well as tumor growth, of HCC and reduced the population of CD133-expressing HCC cells compared with Sorafenib treatment alone, thereby providing a new treatment regime for patients with HCC [97]. All the above-mentioned evidence supports that Notch/HH signaling-based nanotherapies can improve the anticancer effect.

**Table 4:** Anticancer effect of Notch and HH signaling-based nanotherapies.

Nanomedicine Name	Drug in Nanomedicine	Delivery System	Target Cancer	Experimental Model	Effect of Nanomedicine on Cancer	Ref.
Silica NPs	γ-Secretase inhibitor	Mesoporous silica NPs functionalized with glucose moieties	Breast cancer	Human MCF7 and MDA-MB-231 breast cancer cell lines	Reduced cancer stem cell population	[90]
PLGA NPs	α-Mangostin	PLGA NPs	Colorectal cancer	Human colorectal cancer (HCT116 and HT29) cell lines	Inhibited EMT, colony formation, cell viability, and induced apoptosis; suppressed Notch signaling pathway leading to inhibition of cancer stem-like cell population and self-renewal capacity	[91]
Planetary ball-milled NPs	Thymoquine	Planetary ball-milled NPs coated with an RNA aptamer, A10	Prostate cancer	Docetaxel-resistant C4-2B-R and LNCaP-R cells with high expression of HH signaling molecules	Inhibited HH signaling pathway, thereby suppressing prostate cancer progression	[92]
NanoHHi	HPI-1	Polymeric NP (PLGA-PEG) encapsulating HPI-1	Medulloblastoma	Allografts derived from Ptch (−/+); p53 (−/−) mouse medulloblastomas; orthotopic Pa03C pancreatic cancer xenografts	Inhibited tumor growth; downregulated mGli1 and HH target genes	[93]
High-density lipoprotein-mimetic NPs (eHNPs)	LDE225	Apolipoprotein A1 and anti-CD15 incorporated eHNPs	Shh subtype of medulloblastoma	DAOY human medulloblastoma cells and PZp53 cells	Reduced cholesterol in Shh MB cells	[94]
Biomimetic high-density lipoprotein NPs	Synthetic HDL NPs	High-density lipoprotein NPs	Medulloblastoma	In vitro studies using medulloblastoma cell lines	Depleted cholesterol in cancer cells; inhibited proliferation and colony formation; depleted cancer stem cell population	[95]
PEI-SNAs	siRNA targeting Gli1	Polyethylenimine-wrapped spherical nucleic acid NPs	Glioblastoma	Glioblastoma U87-MG cell lines	Silenced tumor-promoting HH pathway genes; decreased glioblastoma cell proliferation; promoted glioblastoma cell senescence; decreased metabolic activity and self-renewal ability of glioblastoma cells; promoted apoptosis	[96]
NanoHHi	Gli1	Polymeric NP-encapsulated delivery system	HCC	In vitro HCC cell lines; in vivo subcutaneous and orthotopic HCC xenografts nude mice	Inhibited invasion and proliferation of HCC cells; suppressed in vivo tumor growth; reduced systemic metastases	[97]

EMT, epithelial–mesenchymal transition; HCC, hepatocellular carcinoma; HH, Hedgehog; NP, nanoparticle; PLGA, poly(lactic-co-glycolic acid); Shh, Sonic hedgehog.

### 2.6. Other Signaling-Based Nanotherapies

Other signaling-based nanotherapies could affect anticancer effects as well. Anticancer effects of other signaling-based nanotherapies are listed in Table 5. Novel elongated-type peanut-shaped gNPs have been tested to evaluate their cytotoxic potential against ovarian SKOV-3 cancer cells [98]. The results revealed that cell viability and the proliferation capability of ovarian cancer cells were decreased because of increased cell apoptosis and autophagy as well as increased reactive oxygen species (ROS) production [98]. RNA NPs containing RNA aptamers binding to the CD133 receptor and inhibiting microRNA-21 have been developed and delivered to breast cancer stem cells [99]. RNA NPs effectively inhibited cancer cell movement and microRNA-21 expression, enhancing the expression of tumor suppressors PTEN and PDCD4 with greater specificity and efficacy [99]. Erlotinib (an inhibitor to epidermal growth factor receptor, EGFR) has been delivered using phospholipase A (PLA)-based NPs [100]. To prevent against the interplay of EGFR with Notch signaling for carcinogenesis, an γ-secretase inhibitor was also enclosed in the core of the NPs with Erlotinib, resulting in effective inhibition of Notch signaling [100]. Because tumors have an acidic microenvironment, NPs can easily target cancer cells by controlling the interleukins (ILs) related to cancer cell resistance at an acidic pH [101]. Because the overexpression of EGFR is noted in 50% of patients with lung cancer and the inhibition of the mitotic regulator polo-like kinase 1 (PLK1) can enhance radiation sensitivity, EGFR-positive NSCLC cells were targeted by the siPLK1-NP [102]. This resulted in the reduced expression of PLK1, which led to cell death, tumor growth reduction, G2/M cell cycle arrest, and extended survival [102]. This indicated that siPLK1-NPs could be an effective targeted therapy that can function as a radiation sensitizer in NSCLC [102].

Previous studies have demonstrated that cytosolic PLA2 is an effective therapeutic molecular target in several human metastatic cancers, including leukemia and breast, prostate, and ovarian cancers [103]. Cytosolic PLA2 has diverse functions, including the biosynthesis of eicosanoids such as prostaglandins and leukotrienes, which are mainly involved in the cytochrome c oxidase (COX) and lipoxygenase pathways [102,104]. Gowda et al. [105] developed a novel PEGylated nanoliposomal delivery system that targeted the cytosolic PLA2 inhibitor arachidonyl trifluoromethyl ketone (ATK). Therefore, this nanoliposomal ATK delivery system can increase circulation time, enhance drug stability, and avoid clearance by the reticuloendothelial system [105]. Because it is less toxic to normal cells than to melanoma cancer cells, nanoliposomal ATK delivery has proven to be remarkable in treating melanoma in preclinical trials [105].

An exosome-based nanoformulation loaded with aspirin can be used as an effective anticancer therapy against breast and colorectal cancer cells [106]. This novel nanoexosome-based drug delivery system improved tumor cell cytotoxicity [106]. It exhibits a higher encapsulation capacity and a better dissolution rate than water-soluble drugs [106]. Nanotherapy with immune checkpoint inhibitors could be a good solution for NSCLC treatment because of its enhanced survival rate, reduced side effects, and stimulation of immune responses against malignancies in patients with NSCLC [107]. Immune checkpoint inhibitors are small molecules that disturb the immune checkpoint signaling pathways, thereby impeding the tumor suppression of immune cells [108]. Zhao et al. [109] showed that Cu-doped gold nanoclusters (CuAuNCs) could be useful for C-X-C motif chemokine receptor 4 (CXCR4)-targeting positron emission tomography imaging as an alternative diagnostic method in cancer biology. Nanomaterials and nanoclusters loaded with chemotherapeutic drugs can provide a new avenue in cancer biology for theragnostic applications because of their advantages, such as accurate and early detection of cancer cells and targeted specificity toward tumor cells [110].

Anti-programmed death ligand 1 (anti-PDL1) therapy reduces locally recurrent and distant cancers [111]. Based on the natural targeting potential of platelet to circulating tumor cells, researchers developed an in situ sprayable chemo-immunotherapy gel that acts as a drug reservoir and releases both anti-PDL1 monoclonal antibody and platelet-derived tiny extracellular vesicles combined with doxorubicin (PexD), an anticancer drug that prevents post-surgery tumor recurrence and spread [112]. Anti-PDL1 antibody and PexD co-encapsulated in a fibrin gel can be sprayed using a dual-cartridge sprayer [113]. Because the released anti-PDL1 antibody effectively blocks the PD1/PDL1 pathway while PexD efficiently stimulates the antitumor immune response by inducing tumor immunogenic cell death, entering the systemic circulation through damaged blood vessels, and attaching to circulating tumor cells, the combined use of anti-PDL1 antibodies and PexD triggers strong T cell immunogenic responses, which ultimately initiate the host’s immunogenic response by inhibiting both metastatic potential postsurgery as well as local tumor recurrence [113]. A radioimmunostimulant and PI3Kγ inhibitor, IPI549, can specifically target myeloid cells and act as a catalase to convert endogenous hydrogen peroxide into oxygen to achieve hypoxia-relieved postoperative radiotherapy [114]. Combined use of IPI549 with anti-PDL1 antibodies increased susceptibility to anti-PDL1 therapy and enhanced radiotherapy-mediated immunogenic cell death by reprogramming the tumor microenvironment into an immunogenic phenotype [114]. Ultimately, this acts as a simple and effective therapeutic strategy to inhibit postsurgical cancer recurrence and metastasis.

**Table 5:** Anticancer effect of other signaling-based nanotherapies.

Nanomedicine Name	Drug in Nanomedicine	Delivery System	Target Cancer	Experimental Model	Effect of Nanomedicine on Cancer	Ref.
AuP NPs	Nanogold	Peanut-shaped gNPs	Ovarian cancer	In vitro study using SKOV-3 cells	Decreased proliferation and viability of ovarian cancer cells; induced autophagy and apoptosis; increased oxidative stress of cancer cells	[98]
RNA NPs	Anti-miR21	Chemically and thermodynamically stable RNA NPs	TNBC	In vivo and in vitro studies using TNBC and breast cancer stem-like cells	Reduced migration of cancer cells; inhibited miR21 expression; upregulated expression of tumor suppressors; efficiently inhibited tumor growth	[99]
CF-EB/DART-dual-loaded NPs	Erlotinib (EB) and gamma-secretase inhibitor (GSI)-DAPT	PLA-based nano-platform	TNBC	In vitro studies using MDA-MB-231 cell line	Enhanced tumor penetration ability of drug; reduced side effects of drugs	[100]
Nanographene sheets and SPION@silica nanospheres	SPION	Nanographene sheets and SPION@silica nanospheres	Breast cancer	In vitro study using MDA-MB 231 cancer cells	Enhanced apoptosis, necrosis, and oxidative stress induction in cancer cells; disrupted cell cycle phases; increased the levels of anticarcinogenic interleukins	[101]
C-siPLK1-NP	Small interfering RNA (siRNA) against PLK1	Cetuximab-conjugated NP	NSCLC	In vitro and in vivo studies using EGFR and NSCLC cells, A549 flank tumors, and an orthotopic lung tumor model	Reduced PLK1 expression; caused cell cycle arrest; induced reduction in tumor growth and cell death	[102]
NanoATK	Arachidonyl trifluoromethyl ketone (ATK)	Nanoliposomal delivery system	Melanoma	Xenograft tumor model	Decreased cellular proliferation, triggered apoptosis, and inhibited melanoma xenograft tumor growth without animal weight loss; inhibited the STAT3, AKT, and cPLA2 pathways	[105]
Nano-amorphous aspirin-loaded exosomes	Aspirin	Exosomes	Breast and colorectal cancers	Human colorectal adenocarcinoma HT29 cell line and human metastatic breast cancer MDA-MB-231 cell line	Enhanced cellular uptake, improved cytotoxicity of aspirin, increased apoptosis and autophagy, eradication of cancer stem cells, efficient delivery to in vivo tumors	[106]
CuAuNCs	AMD3100 (also known as Plerixafor)	Gold nanoclusters	Breast cancer and lung metastasis	Mouse 4T1 orthotopic breast cancer model	Sensitive and accurate detection of CXCR4 in early-stage cancers; accurate imaging for early detection of breast cancer	[109]
PexD	Doxorubicin and Adp-L1	Sprayable bioresponsive gel	Melanoma	B16-F10 tumor-bearing mice	Inhibited local tumor recurrence and metastasis, induced tumor immunogenic cell death, promoted antitumor immune response, tracked and eliminated circulating tumor cells, impaired PD-1/PD-L1 pathway, restored the tumor-killing effect of cytotoxic T cells, improved tumor immune microenvironment	[113]
IPI549@HMP	IPI549 (PI3Kγ inhibitor)	PEGylated HMnO_2_ (HMP)-bridged radioimmunotherapy nanoplatform	Cancer recurrence after surgery	Experimental model demonstrating the genomic landscape shaped by surgical resection and the effects on the tumor microenvironment	Suppressed/eradicated local residual and distant tumors and elicited strong immune memory effects to resist tumor rechallenge	[114]

AKT, AKT serine/threonine kinase 1; gNP, gold NP; NP, nanoparticle; NSCLC, non-small cell lung cancer; PexD, platelet-derived tiny extracellular vesicles combined with doxorubicin; PLA, phospholipase A; PLK1, polo-like kinase 1; TNBC, triple-negative breast cancer.

## 3. Approved Nanomedicines Currently Available for Anticancer Therapy

Approved nanomedicines currently available for anticancer therapy are listed in Table 6. Several types of nanomedicines have been approved by official regulatory institutions including the Food and Drug Administration (FDA) for cancer treatment, leveraging the unique properties of NPs to enhance drug efficacy and delivery [115,116,117]. Doxil^®^ (Doxorubicin Liposome) is a liposomal formulation of doxorubicin, used in treating various cancers including ovarian cancer and multiple myeloma. The liposome helps to reduce the cardiotoxicity associated with doxorubicin. Abraxane^®^ (Paclitaxel Albumin-bound) uses albumin NPs in delivering paclitaxel, a chemotherapy drug. It is used to treat breast cancer, NSCLC, and pancreatic cancer. Irinotecan Liposome Onivyde^®^ is used to treat metastatic pancreatic cancer. The liposome helps to improve the drug’s pharmacokinetics and reduce side effects. Vyxeos^®^ (Daunorubicin and Cytarabine Liposome) contains daunorubicin and cytarabine in a liposomal formulation and it is used to treat acute myeloid leukemia. The liposome allows for a more controlled release of the drugs. These nanomedicines represent significant advancements in cancer therapy, offering improved targeting and reduced toxicity compared to traditional formulations.

## 4. Phytochemicals Used in Anticancer Therapy

Patients with cancer who have undergone chemotherapy had higher levels of inflammation compared with their healthy counterparts [118]. For this reason, the development of anticancer drugs with anti-inflammatory properties and low toxicity could be a potential therapeutic strategy in treating patients with cancer [14]. Because phytochemicals could be used for anti-inflammatory purposes to aid anticancer application [11,12,13], natural phytochemicals that could be used as anticancer drugs are summarized in this Section. Natural phytochemicals used in anticancer therapy are listed in Table 7.

### 4.1. Phytochemicals against Inflammatory Microenvironment in Cancer

Scutellarin, a flavone glucuronide, has been extracted from *Erigeron breviscapus*, a traditional Chinese medicine plant [119]. It can inhibit the production of proinflammatory mediators by inhibiting the MAPK and I-kappaB kinase (IKK)-dependent nuclear factor-kappa B (NFκB) signaling pathway [112]. TQ is an important constituent of black cumin seed oil from *Nigella sativa* [120]. TQ can inhibit NFκB-dependent neuroinflammation in BV2 microglia via activating the antioxidant response element (ARE)/nuclear erythroid 2 related factor 2 (Nrf2) antioxidant pathway [120]. Oxyresveratrol is a polyphenolic molecule present in various plants, including *Artocarpus lakoocha* [121]. It exerts anti-inflammatory effects in IL-1β-induced human microglial clone 3 cells by inhibiting ERKs on MAPK signaling cascades and the AKT on PI3K signaling cascades, indicating that oxyresveratrol could be an effective pharmacologic agent to treat neuroinflammation in microglia [121]. Terpenoids extracted from *Abies holophylla* exert neuroprotective and anti-inflammatory effects via increasing nerve growth factor production and decreasing nitrite production through the inhibition of JNK phosphorylation, thereby inhibiting the secretion of proinflammatory cytokines such as IL-1β, IL-6, tumor necrosis factor (TNF), and prostaglandin E2, and effectively decreasing neuroinflammation in microglial cells [122]. Curcumin, a phytochemical extracted from *Curcuma longa*, possesses antioxidant, anticancer, and anti-inflammatory effects [11]. Curcumin can decrease neuroinflammation post-subarachnoid hemorrhage by inhibiting the toll-like receptor/NFκB signaling pathway and sequentially a shift of microglia M1 phenotype to M2, which promotes tumor survival [123,124]. Moringin, isolated from *Moringa oleifera* seeds, effectively normalized Wnt/β-catenin signaling in mice with autoimmune encephalomyelitis [125]. Moringin can upregulate β-catenin and inhibit glycogen synthase kinase-3, which regulates FoxP3 and CD4 expression in T cell activation, inhibiting COX-2, IL-6, and IL-1β, decreasing apoptosis, and increasing expression of antioxidant Nrf2 in mice with autoimmune encephalomyelitis [125]. Hesperetin, a phytochemical, can effectively inhibit nitric oxide, decrease expression of IL-1β, IL-6, and MAPK, downregulate ERK1/2 phosphorylation, suppress astrocyte and microglial cell activation, and ultimately decrease neuroinflammation in BV-2 microglial cells [126].

### 4.2. Phytochemicals against Postsurgical Recurrence of Cancer and Metastasis

Rottlerin is a natural polyphenol compound [127]. It can inhibit metastasis-related MMPs by inhibiting protein kinase C (PKC)-mediated ROS, inactivating ERK1/2, and suppressing the AP-1/c-Fos signaling pathway, which suppresses astrocyte migration in phorbol-12-myristate-13-acetate-induced rats [127]. Genistein (a soy-derived isoflavone) could be used to boost the inhibitory role of cisplatin widely used to treat HCC to protect against tumor recurrence and metastasis following curative hepatectomy [12,13]. Some experimental evidence has shown that the combined use of genistein with cisplatin might lower the dose requirement of cisplatin as well as improve anticancer activity in various malignancies, including lung, prostate, pancreatic, and breast cancers [128]. Furthermore, various combinations of drugs showed greater inhibitory effects against cancers than the use of individual drugs alone [13].

## 5. Application of Nanotechnology and Phytochemicals in Clinical Trials for Anticancer Therapy

Because of the previously mentioned strengths of nanotechnology and phytochemicals, the combined use of nanotechnology with phytochemicals has been applied to clinical trials for anticancer therapy, as shown in Table 8. Patients treated with the Nano Swarna Bhasma (NSB) drug showed 100% clinical benefit compared to patients treated without NSB, indicating the clinical role of NSB [129]. CRLX101, a cyclodextrin-containing polymer NP loaded with camptothecin, was prescribed to patients with esophageal or gastrointestinal cancers [15,130]. In a phase II clinical trial of CRLX101, it downregulated tumor biomarkers in gastric, gastroesophageal, and esophageal cancers [130]. The safety of camptothecin was checked in patients with advanced rectal carcinoma [15]. This phytochemical not only resulted in the downstaging of rectal cancer but did not induce any severe side effects among the patients treated [15]. A cyclodextrin-containing polymer loaded with docetaxel led to the stable condition of patients with prostate or breast adenocarcinoma with a 19.4% clinical benefit rate [131]. Moreover, this nanoformulated drug exhibited some pharmacokinetic advantages over docetaxel, including longer retention of drug in plasma, slower clearance, and controlled release rate of docetaxel from the carriers [131]. When another drug Pm-Pac, polymeric micellar NPs conjugated with phytochemical paclitaxel, was used in treating patients with advanced NSCLC, it significantly increased the overall and progression-free survivals of NSCLC patients without pleural metastasis [132]. When another phytochemical, ursolic acid, was loaded into nanoliposomes (UANL), it did not accumulate in the body and showed no adverse effects when it was treated at 37 mg/m^2^ of UANL [133]. Overall, the combined application of nanotechnology and phytochemicals can give many benefits in relation to higher treatment efficacy, lower side effects, a more stable condition, and prolonged overall survival after treatment of patients with cancer.

## 6. Current Challenges and Opportunities for Future Nanotherapeutic Strategies

Generally, cancer shows uncontrolled cell development because of the deactivation of tumor suppressors or activation of oncogenes, dysregulated cell cycling, and metastatic properties, and it is a main cause of mortality worldwide [134]. Although surgery tends to be the primary treatment option for many solid cancers, cancer surgery is still a risk factor for metastatic diseases and recurrence [3]. Although a variety of medications has been adopted for the postsurgical care of patients with cancer [4], conventional medicines have shown major challenges such as drug resistance, a high level of drug toxicity, and different drug responses due to tumor heterogeneity [5,6]. Nanocarriers in nanomedicine could be modulated and thus nanotechnology-based therapeutic formulations could effectively overcome the challenges faced by conventional treatment methods [50,51,52,53,54,55,56,57,58,59,60,61,62,63,64,65,66,67,68,69,70,71,72,73,74,75,76,77,78,79,80,81,82,83,84,85,86,87,88,89,90,91,92,93,94,95,96,97,98,99,100,101,102,103,104,105,106,107,108,109,110,111,112,113,114]. In relation to anticancer drugs in nanomedicine, the proteins against several carcinogenesis signaling mechanisms, including the TGF-β, MAPK, PI3K, Wnt, PARP, Notch, and HH signaling pathways, should be considered in anticancer therapy [10]. Because natural phytochemicals can support reducing carcinogenesis-related inflammation, they should also be considered in the development of anticancer drugs [11,12,13]. As proven in anticancer nanodrugs approved by official regulatory institutions such as the FDA, nanomedicine can provide better anticancer drug efficacy, overcoming major constraints in conventional chemotherapy such as poor solubility, many side effects, and low bioavailability [5,9,115,116,117]. In addition, during anticancer therapy, nanomedicine has a better treatment potential because it can deliver chemotherapeutic agents to specific tumor sites better [6]. Moreover, nanomedicine helps conventional medicines overcome their major challenges such as drug resistance, a high level of drug toxicity, and different drug responses [6]. In particular, the combined use of nanotechnology with natural phytochemicals can enhance tumor targeting and increase the efficacy of anticancer agents with better solubility and bioavailability and reduced side effects [135,136]. Furthermore, nanomedicine can transfer multiple materials, including DNA, RNA, fluorescence agents, and so on, as well as drugs, to tumor sites specifically in a controlled manner [137]. The control of the continuous secretion of anticancer drugs using NPs by regulated light intensity and the prevention against phagocytic clearance of NPs by their surface modifications could give better benefits in the treatment of patients with cancer [138,139]. In addition, NPs allow imaging for the detection, diagnosis, and monitoring of treatment outcomes, as well as delivery of therapy [140]. Therefore, in the future, the anticancer effect of various NPs should be evaluated in clinical trials to consider their safety.

## Figures and Tables

**Table 1:** Anticancer effect of TGF-β signaling-based nanotherapies.

Nanomedicine Name	Drug in Nanomedicine	Delivery System	Target Cancer	Experimental Model	Effect of Nanomedicine on Cancer	Ref.
ECO/siRNA NPs	β3 Integrin siRNA	Lipid ECO-based NPs	TNBC	MDA-MB-231 cell line and NME cell line	Silenced the expression of Integrin β3; lessened TGF-β mediated epithelial-mesenchymal transition and metastasis	[50]
Poly-N-(2-hydroxypropyl) methacrylamide-coated W-LPNs (S/C-pW-LPNs)	Silibinin and cryptotanshinone	Poly-N-(2-hydroxypropyl) methylacrylamide-coated wheat germ agglutinin-modified lipid-polymer hybrid NPs	Breast cancer	4T1 breast cancer cells; 4T1 tumor-bearing nude mouse model	Increased 4T1 cell toxicity; inhibited cell invasion and migration; reduced tumor progression and metastasis to the lungs	[51]
ZP6	Zinc oxide	Zp6 Capped with aminopolysiloxane	Retinal degenerative diseases	Murine photoreceptor-derived 661W cell line	Formation of apoptotic bodies; disruption of cell cycle; disruption of intracellular calcium homeostasis and increase in oxidative stress; reduction in the expression of TGF-β and matrix metalloprotease 9	[52]
PLG(Ag) NPs	TGF-β and OVA peptide	PLGA NPs	Multiple sclerosis and autoimmune encephalomyelitis	Mouse model for multiple sclerosis and autoimmune encephalomyelitis	Reduced inflammation in bone marrow-derived dendritic cells; induced regulatory T cells; reduced disease severity	[53]
MSNP-PEI-PEG	SiHSP47	Polyethylenimine and polyethylene glycol coating on mesoporous silica NP	Fibrotic disease (scleroderma)	TGF-β stimulated fibroblasts; bleomycin-induced scleroderma mouse model	Reduced HSP47 protein expression; reduced NADPH oxidase 4 levels; reduced pro-fibrotic markers	[54]

HSP47, heat shock protein 47; NP, nanoparticle; PLGA, poly(lactic-co-glycolic acid); TGF-β, transforming growth factor-beta; TNBC, triple-negative breast cancer.

**Table 3:** PARP signaling-based nanotherapies.

Nanomedicine Name	Drug in Nanomedicine	Delivery System	Target Cancer	Experimental Model	Effect of Nanomedicine on Cancer	Ref.
NanoTalazoparib	Talazoparib	Bilayer nano-liposome	*BRCA*-mutated metastatic breast cancer	*BRCA*-deficient mice	Induced DNA damage, cell cycle arrest, and inhibition of cell proliferation in tumors; modulated immune cell populations; decreased myeloid-derived suppressor cells in tumors and spleen	[78]
NanoOlaparib	Olaparib	Lipid-based injectable nanoformulation	Advanced prostate cancer	*PTEN*/*p53*-deficient mouse with prostate cancer	Made tumors more radiation-sensitive; caused significant tumor growth inhibition	[79]
Nanoemulsion encapsulated PARPi-FL	PARPi-FL (fluorescently labeled sensor for Olaparib)	Nanoemulsion	Small cell lung cancer	Subcutaneous xenografts of small cell lung cancer	Increased blood half-life; improved delineation of small cell lung cancer xenografts	[80]
SMI#9-GNP	SMI#9	gNPs	TNBC	Cell culture models of TNBC	Induced cytotoxicity in mesenchymal TNBC cells; enhanced cisplatin sensitivity when combined with cisplatin; selectively induced cell death through mitochondrial dysfunction and PARP1 stabilization/hyperactivation	[82]
Liposomal NPs	Cisplatin and PARP inhibitors	Liposomal NPs with a terminal hyaluronic acid layer	Ovarian cancer	Luciferase and CD44-expressing orthotopic OVCAR8 xenograft nude mice	Moderated systemic toxicity; reduced tumor metastasis; extended survival	[83]
Lipidoids	siRNA targeting PARP1 (siParp1)	Lipidoids for delivering siRNA	Ovarian cancer	Mouse models of ovarian cancer	Inhibited cell growth, induced apoptosis in *BRCA1*-deficient cells, extended survival in mice with ovarian cancer cells	[84]
PLGA NPs co-encapsulating methylene blue	Veliparib	PLGA NPs	Melanoma	In vitro assays using B16F10-Nex2 cells	Decreased cell viability	[85]
gNP-CALNN	Linalool	gNPs capped with glutathione and conjugated with a CALNN peptide	Ovarian cancer	In vitro assays using SKOV-3 ovarian cancer cells	Induced apoptosis of ovarian cancer cells via activating caspase-8 and apoptosis-associated proteins	[86]

gNP, gold NP; NP, nanoparticle; PARP, poly(ADP-ribose) polymerase; PLGA, poly(lactic-co-glycolic acid); TNBC, triple-negative breast cancer.

**Table 6:** Approved nanomedicines currently available for anticancer therapy [115,116,117].

Institute (Approval Year)	Product	Company	Drug in Nanomedicine	Delivery System	Target Cancer
FDA (1994, 2006)	Oncaspar	Enzon-Sigma-tau	Pegaspargase/L-asparaginase	Polymer conjugate	Acute lymphoblastic leukemia
FDA (1996)	DaunoXome	Gilead Sciences	Daunorubicin	Liposome	Kaposi’s sarcoma
FDA (1999)	DepoCyt	Pacira Pharmaceuticals	Cytarabine	Liposome	Neoplastic meningitis
FDA (2005)	Abraxane	Abraxis/Celgene	Paclitaxel	NP-bound albumin	Breast and pancreatic cancer, NSCLC
FDA (2012)	Marqibo	Talon Therapeutics/Spectrum Pharmaceuticals	Vincristine	Liposome	Acute lymphoblastic leukemia
FDA (2015)	Onivyde	Merrimack Pharma	Irinotecan	Liposome	Pancreatic cancer, colorectal cancer
FDA (1995, 1999, 2007), EMA (1996, 2000), Taiwan (1998)	Doxil, Caelyx, Myocet, and Lipo-Dox	Johnson and Johnson, Schering-Plough, Teva UK, and TTY Biopharm	Doxorubicin	Liposome	Metastatic breast cancer, ovarian cancer, Kaposi’s sarcoma, multiple myeloma
FDA (2017) EMA (2018)	Vyxeos	Celator/Jazz Pharma	Daunorubicin/Cytarabine	Liposome	Acute myeloid leukemia
EMA (2009)	Mepact	Takeda Pharmaceuticals	Mifamurtide MTP-PE	Liposome	Osteosarcoma
EMA (2010, 2013)	NanoTherm	MagForce Nanotechnologies AG	Thermal ablation using a magnetic field	Iron oxide nanoparticles	Glioblastoma, prostate, and pancreatic cancer
EMA (2019)	Hensify (NBTXR3)	Nanobiotix	No drug with radiotherapy	Hafnium oxide nanoparticle	Locally advanced soft tissue sarcoma (STS)
EMA (2019)	Pazenir	Ratiopharm GmbH	Paclitaxel	NP-bound albumin	Metastatic breast cancer, metastatic adenocarcinoma of the pancreas, NSCLC
Republic of Korea (2007)	Genexol-PM	Samyang Biopharmaceuticals	Paclitaxel	PEG-PLA polymeric micelle	Breast, lung, and ovarian cancer

EMA, European Medicines Agency, FDA, US Food and Drug Administration, NP, nanoparticle; NSCLC, non-small cell lung cancer; PEG, polyethylene glycol; PLA, phospholipase A.

**Table 7:** Phytochemicals used in anticancer therapy.

Source of Phytochemical	Chemical Structure	Experimental Model	Action Mechanism of Phytochemical	Ref.
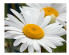 Erigeron breviscapus	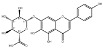 Scutellarin	Lipopolysaccharide-induced BV-2 microglial cells	Inhibit the production of proinflammatory mediators by inhibiting MAPK and I-kappa B kinase (IKK)-dependent NFκB signaling pathway	[119]
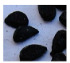 Black cumin seed of Nigella sativa	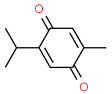 Thymoquinone	Lipopolysaccharide-induced BV-2 microglial cells	Inhibit NFκB-dependent neuroinflammation in BV2 microglia via activating the antioxidant response element (ARE)/nuclear erythroid 2 related factor 2 (Nrf2) antioxidant pathway	[120]
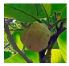 Artocarpus lakoocha	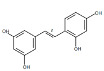 Oxyresveratrol	Human microglial cells	Exerts anti-inflammatory roles in IL-1β-induced human microglial clone 3 cells by inhibiting extracellular signal-regulated kinases (ERKs) on MAPK signaling cascades and the AKT serine/threonine kinase on PI3K signaling cascades	[121]
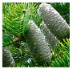 Abies holophylla	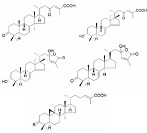 Terpenoids	Lipopolysaccharide-activated BV2 murine microglial cells	Exert neuroprotective and anti-inflammatory effects by decreasing production of nitrite and increasing the production of nerve growth factor through the inhibition of JNK phosphorylation, thereby inhibiting the secretion of proinflammatory cytokines such as IL-1β, IL-6, TNF, and prostaglandin E2, and effectively decreasing neuroinflammation	[122]
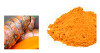 Curcuma longa	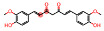 Curcumin	Head and neck squamous carcinoma cells, TLR4(−/−) or wild type of subarachnoid hemorrhage-induced mice model	Possesses antioxidant, anticancer, and anti-inflammatory effects Decrease neuroinflammation post-subarachnoid hemorrhage by inhibiting toll-like receptor/NFκB signaling pathway and sequentially a shift of microglia M1 phenotype to M2, which promotes tumor survival	[11,123,124]
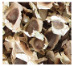 Moringa oleifera seed	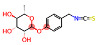 Moringin	Autoimmune encephalomyelitis mice model	Normalize the Wnt/β-catenin signaling pathway Upregulate β-catenin and inhibit glycogen synthase kinase-3, which leads to the regulation of FoxP3 and CD4 expression in T cell activation, inhibition of COX-2, IL-6, and IL-1β, decreased apoptosis, and increased expression of antioxidant Nrf2	[125]
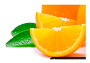 Citrus fruits	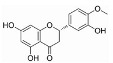 Hesperetin	Lipopolysaccharide-stimulated BV-2 microglial cells	Inhibit nitric oxide, decrease expression of IL-1β, IL-6, and MAPK, downregulate ERK1/2 phosphorylation, suppress astrocyte and microglial cell activation, and ultimately decrease neuroinflammation	[126]
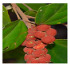 Mallotus philippinensis	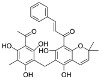 Rottlerin	Phorbol 12-myristate 13-acetate (PMA)-induced rat brain astrocytes	Inhibit metastasis-related matrix metalloproteases by inhibiting PKC-mediated ROS, inactivating ERK1/2, and suppressing the AP-1/c-Fos signaling pathway, which suppresses astrocyte migration in phorbol-12-myristate-13-acetate-induced rats	[127]
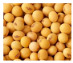 Soy	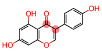 Genistein	Hepatectomy model of nude mice bearing human hepatocellular carcinoma xenografts, colon HT-29, and breast MCF-7 cancer cells	Boost the inhibitory role of cisplatin Protect against tumor metastasis and recurrence following curative hepatectomy Lower the dose requirement of cisplatin as well as improving anticancer activity in many malignancies, including prostate, lung, breast, and pancreatic cancers	[12,13,128]

**Table 8:** Combined application of nanotechnology with phytochemicals in clinical trials for anticancer therapy.

Nanomedicine Name	Phytochemical in Nanomedicine	Delivery System	Target Cancer	Target Population	Effect of Nanomedicine on Anticancer Therapy	Treatment Stage	Ref.
NSB	Mangiferrin	gNPs	Breast cancer	Female patients with stage IIIA or IIIB of breast carcinoma	Patients who received nanomedicine alongside the standard care had a 100% clinical benefit rate when compared to those who only received the standard care; only one patient showed severe adverse effects	Pilot preclinical trial	[129]
CRLX101	Camptothecin	Cyclodextrin-containing polymer NPs	Gastric, gastroesophageal or esophageal cancer	Patients with gastroesophageal, esophageal, or gastric cancer who are on at least one line of systemic therapy	Downregulation of tumor indicators such as topoisomerase I and carbonic anhydrase IX	Phase II clinical trial	[130]
CRLX101	Camptothecin	Cyclodextrin-containing polymer NPs	Rectal cancer	Adult patients with T3–4N0 or T1–4N+ of rectal cancer	Asymptomatic lymphopenia was recorded with a high dose of the drug; downstaging occurred in 69% of patients; pathologic complete response was achieved in 19% of patients overall and 33% of patients at the weekly maximum tolerated dose	Phase Ib/II clinical trial	[15]
CRLX301	Docetaxel	Cyclodextrin-containing polymers	Advanced or metastatic prostate and breast adenocarcinoma	Patients with prostate or breast adenocarcinoma	Found 19.4% of clinical benefit rate; presented some pharmacokinetic advantages over docetaxel	Phase I/IIa clinical trial	[131]
Pm-Pac	Paclitaxel	Polymeric micellar NPs	NSCLC	Patients with advanced NSCLC without pleural metastasis	Increased progression-free survival and overall survival of patients	Phase III clinical trial	[132]
UANL	Ursolic acid	Nanoliposomes	Advanced solid tumors including non-Hodgkin’s lymphoma, Hodgkin’s lymphoma, Hepatoma, and gastric cancer	Healthy volunteers and patients with advanced solid tumors	They tested only pharmacokinetic parameters and safety; no accumulation with repeated doses of UANL; no adverse event in patients who received 37 mg/m^2^ of UANL; no adverse effect after the provision of the larger doses	Phase I clinical trial	[133]

gNP, gold NP; NP, nanoparticle; NSCLC, non-small cell lung cancer; UANL, ursolic acid-loaded nanoliposomes.
